# Adenoviral Vectors Armed with PAPILLOMAVIRUs Oncogene Specific CRISPR/Cas9 Kill Human-Papillomavirus-Induced Cervical Cancer Cells

**DOI:** 10.3390/cancers12071934

**Published:** 2020-07-17

**Authors:** Eric Ehrke-Schulz, Sonja Heinemann, Lukas Schulte, Maren Schiwon, Anja Ehrhardt

**Affiliations:** Institute for Virology and Microbiology, Department for Human Medicine, Faculty of Health, Center for Biomedical Education and Research (ZBAF), Witten/Herdecke University, Stockumer Street 10, 58453 Witten, Germany; Eric.Ehrke-Schulz@uni-wh.de (E.E.-S.); Sonja.Heinemann@uni-wh.de (S.H.); Lukas.Schulte@uni-wh.de (L.S.); m.schiwon@gmx.de (M.S.)

**Keywords:** papillomavirus, HPV, CRISPR, gene therapy, viral vector, adenovirus

## Abstract

Human papillomaviruses (HPV) cause malignant epithelial cancers including cervical carcinoma, non-melanoma skin and head and neck cancer. They drive tumor development through the expression of their oncoproteins E6 and E7. Designer nucleases were shown to be efficient to specifically destroy HPV16 and HPV18 oncogenes to induce cell cycle arrest and apoptosis. Here, we used high-capacity adenoviral vectors (HCAdVs) expressing the complete CRISPR/Cas9 machinery specific for HPV18-E6 or HPV16-E6. Cervical cancer cell lines SiHa and CaSki containing HPV16 and HeLa cells containing HPV18 genomes integrated into the cellular genome, as well as HPV-negative cancer cells were transduced with HPV-type-specific CRISPR-HCAdV. Upon adenoviral delivery, the expression of HPV-type-specific CRISPR/Cas9 resulted in decreased cell viability of HPV-positive cervical cancer cell lines, whereas HPV-negative cells were unaffected. Transduced cervical cancer cells showed increased apoptosis induction and decreased proliferation compared to untreated or HPV negative control cells. This suggests that HCAdV can serve as HPV-specific cancer gene therapeutic agents when armed with HPV-type-specific CRISPR/Cas9. Based on the versatility of the CRISPR/Cas9 system, we anticipate that our approach can contribute to personalized treatment options specific for the respective HPV type present in each individual tumor.

## 1. Introduction

Human papillomaviruses (HPV) are small non-enveloped epitheliotropic DNA viruses with a circular genome comprised of approximately 8000 base pairs (bp). So called high-risk HPV are responsible for the development of malignant cervical carcinoma and their precursor lesions cervical intraepithelial neoplasia (CIN) [1]. Currently over 200 different HPV types are known. Due to their carcinogenic properties, especially HPV16, 18 and 31 (but also 33, 35, 39, 45, 51, 52, 56, 58, 59, 68, 73, and 82) are classified as high-risk HPV types [2]. HPV DNA was also found in oropharyngeal carcinomas summarized as head and neck cancer (HNC) and are therefore regarded as important carcinogens [3]. Furthermore, HPV are a cofactor in the development of dermatologic malignancies, such as Squamous cell carcinoma (SCC) and basal cell carcinoma (BCC) of the skin summarized as non-melanoma skin cancer (NMSC), and their precursor lesions, actinic keratosis (AK) [4]. Despite the availability of a protective vaccination against high-risk HPV, not all girls and young women (as well as boys and young men) are vaccinated. Therefore, it is likely, that HPV-associated tumors represent a continuous health care burden. HPV-associated tumors such as cervical carcinoma, NMSC and HNC can be removed surgically, but a complete detection and removal of tumor tissue including possible precursor lesions in the immediate vicinity is difficult as non-infected regions, HPV infected but non transformed regions, precursor lesions and invasive carcinomas are located in close proximity of an epithelial area [5]. The conventional cancer therapies such as radiation or chemotherapy in concert with surgical resection increase the chances of recovery but are associated with strong side effects. Therefore, the development and testing of alternative treatment strategies are desirable.

In the context of cervical infection, the HPV genome can be integrated into the chromosomal DNA of basal epithelial cells. During this process, the episomal, circular HPV genome is linearized within the HPV E2 gene, leading to a loss of E2 function. As the HPV E2 protein negatively regulates HPV E6 and E7 gene expression, the loss of HPV E2 function leads to unregulated overexpression of the HPV oncogenes E6 and E7, that mediate transformation of the host cell by inducing cell proliferation, bypassing cell cycle control and inhibition of apoptosis. The HPV E6 protein interacts with the tumor suppressor protein p53, causes its degradation and thus prevents cell cycle control and the initiation of apoptosis [6]. The HPV E7 protein interacts with the retinoblastoma protein pRB and causes the release of the pRB-bound E2F transcription factor. E2F subsequently induces the expression of genes that induce cell cycle progression [7,8]. In recent years, several approaches have been described to induce cell cycle arrest and induction of apoptosis in HPV positive cancer cells, either by specifically inhibiting HPV oncoprotein interactions using intracellular antibodies [9] or peptides such as HPV E7 antagonist [10], E6-binding aptamer [11], E6-AP mimetic epitope [12], or RNA molecules like Anti-E6 ribozyme [13]. Moreover, downregulating HPV oncogene expression using RNA interference [14,15,16,17,18,19,20,21] has been shown to be a promising treatment option for HPV induced tumors. However, only the most recent studies tried to translate these in vitro findings toward in vivo applications using non-viral RNA delivery or AAV-mediated viral delivery.

Designer nucleases such as zinc finger nucleases (ZFN), transcription activator-like effector nuclease (TALEN) and especially clustered regularly interspaced short palindromic repeats (CRISPR/Cas9) are highly efficient customizable molecular scissors for sequence-specific induction of in/del mutations at the DNA-target site. This gene disruption strategy has been applied in antiviral approaches against DNA viruses such as Hepatis B virus (HBV) [22,23,24]. In the context of HPV-related cancer, it was reported that cleavage of the HPV18 origin of replication by artificial zinc-finger proteins fused to a bacterial nuclease inhibited HPV DNA replication [25]. CRISPR/Cas9 mediated inactivation of HPV18- and HPV16 E6 or E7 resulted in the induction of p53 or pRb, leading to cell cycle arrest and cell death [26]. CRISPR/Cas9 mediated disruption of HPV E7 open reading frame (orf) alone induced apoptosis and growth inhibition in HPV16 positive cervical cancer cells [27] and HPV6/11 E7-expressing keratinocytes [28]. Moreover, it was shown that a CRISPR/Cas9 approach to specifically destroy the HPV16 early promoter, HPV16 E6, or HPV16 E7 coding regions lead to decreased cell viability and increased apoptosis induction and decreased growth of SiHa cell-derived tumors implants in BALB/C nude mice [29]. Interestingly cleavage of HPV16 E6 alone was sufficient to induce apoptosis and growth inhibition of HPV16-positive cells [30].

However, delivery approaches to enable comprehensive in preclinical in vivo studies are rare. A recent study showed that non-viral CRISPR/Cas9 delivery using PEGylated liposomes resulted in tumor elimination in vivo [31]. Numerous publications reported the CRISPR/Cas9 delivery using AAV co-transduction approaches. However, these vectors are rather small and do not allow to deliver all CRISPR/Cas9 components including one or several guide RNAs within one vector. Even though CRISPR/Cas9 is particularly suitable for arming conditionally replicating Adenoviruses (CRAdVs)/oncolytic Adenoviruses [32,33,34], the potential of viral delivery of HPV-specific CRISPR/Cas9 was not fully exploited. Adenoviruses (AdV) infect a great variety of different cells types and tissues and can enter quiescent as well as dividing cells. Another benefit of the AdV vector system arises from the non-integrating, episomal persistence of the viral genome [35]. As AdV do not integrate their genome into host cell chromosomes genotoxicity related to insertion into transcribed genomic loci is circumvented. To translate the non-viral CRISPR approaches of the preceding studies into an all-in-one viral vector delivery approach to enable future in vivo studies (high-capacity adenoviral vectors (HCAdV)) offer several benefits. In HCAdV genomes all viral coding sequences have been removed and only the inverted terminal repeats (ITR) and the packaging signal that is necessary for vector genome replication and efficient genome packaging are still present [36,37]. The HCAdV packaging capacity of up to 35 kb allows transporting the whole CRISPR/Cas9 machinery including several gRNAs [38]. As HCAdV do not express AdV genes, they are regarded as less immunogenic than early generation AdV vectors [39,40]. Nevertheless, production of HCAdV are time and work intensive when compared with Lentivirus- or AAV-vector platforms, hampering their exploration for specific applications.

In this study, we aim to exploit the advantages of the HCAdV platform and constructed HCAdVs expressing the complete CRISPR/Cas9 machinery including Cas9 and a gRNA specific for HPV16-E6 [29] or HPV18E6 [26,38] within a single vector. These non-replicating HCAdV vectors express the HPV E6 specific CRISPR/Cas9 and destroy the respective E6 oncogene. Upon vector transduction of HPV positive cervical carcinoma cell lines HeLa, CaSki, and SiHa and HPV-negative lung carcinoma cell line A549, we examined the potential of the HPV E6 specific CRISPR/Cas9 expressing HCAdV to mediate apoptosis induction, inhibition of tumor cell growth, and increase of tumor cell death.

## 2. Results

### 2.1. HPV Oncogene Specific CRISPR Expressed from HCAdVs Efficiently Disrupts PV E6 Oncogenes

**Figure 1:**
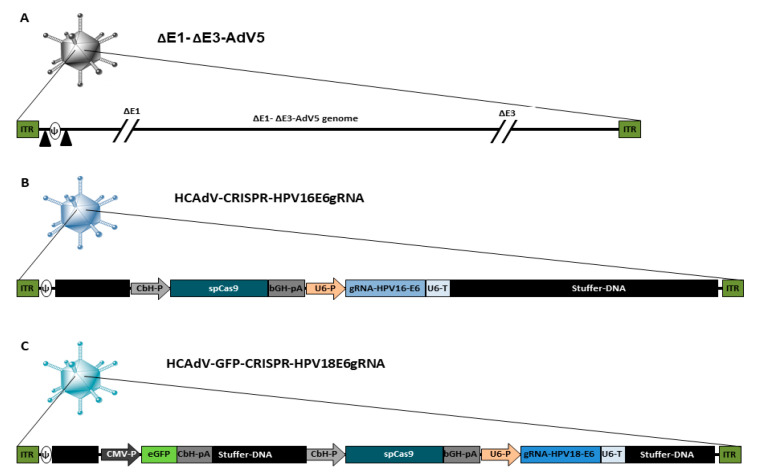
Schematic presentation of the genome organization of the vectors used in this study. (**A**) ΔE1-ΔE3-AdV5 contains a wild type AdV5 genome lacking the E1- and E3 gene, the packaging signal is flanked by loxP sites indicated by black triangles. (**B**) HCAdV-CRISPR-HPV16E6gRNA contains a spCas9-gene from Staphylococcus pyogenes controlled by a CbH-promoter and a bGH-pA and a gRNA expression cassette with specificity toward HPV16-E6 controlled by a human U6-promoter and a U6-terminator sequence. (**C**) HCAdV-CRISPR-HPV18E6gRNA contains a spCas9-gene controlled by a CbH-promoter and a bGH-pA and a gRNA expression cassette with specificity towards HPV18-E6 controlled by a human U6-Promoter and a U6-terminator sequence. It also contains an enhanced GFP transgene expression cassette controlled by a CMV promoter and a CbH-pA. CbH-P, chicken β actin hybrid promoter; bGH-pA, bovine growth hormone polyadenylation signal; gRNA, guide RNA; U6-P, human U6- small nuclear RNA promoter; U6-pA, U6- small nuclear RNA polyadenylation signal; CMV-P, Cytomegalovirus promoter, CbH-pA chicken β actin hybrid polyadenylation signal; ITR, inverted terminal repeat; Ψ, AdV5 packaging signal.

A schematic overview of the HCAdV vector genomes used in this study is presented in Figure 1. As a control virus, we used the E1- and E3-deleted first generation adenoviral vector ΔE1-ΔE3-AdV5, which can replicate in HPV-positive cancer cells. Furthermore, two HCAdVs-encoding CRISPR/Cas9 and gRNA against the HPV16 oncogene E6 (HCAdV-CRISPR-HPV16E6gRNA) and the HPV18 oncogene E6 (HCAdV-GFP-CRISPR-HPV18E6gRNA) were generated (Figure 1).

**Figure 2:**
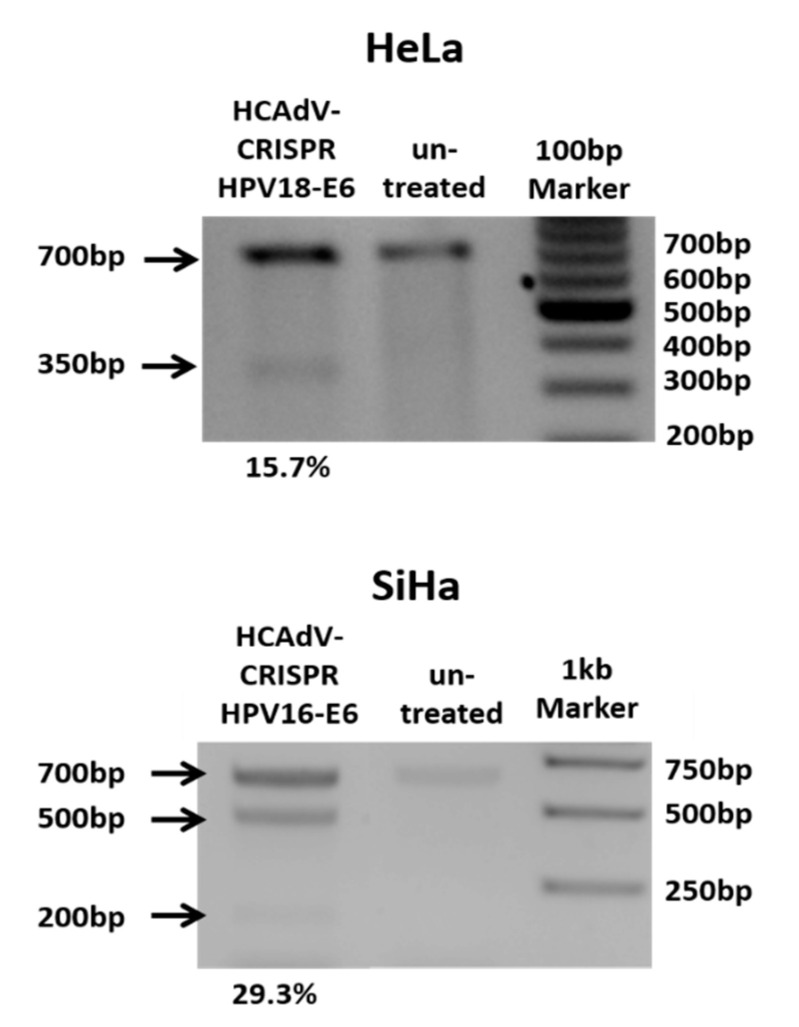
Heteroduplex based mutation detection of HPV16-E6 and HPV18-E6 after treatment of SiHa or HeLa cells at multiplicity of infection (MOI) 500 per cell. 48 h post transduction the T7E1-assay showed efficient mutagenesis of HPV-E6-genes of cells treated with HPV specific CRISPR HCAdV compared to cells that were not transduced. Arrows indicate the expected size of HPV-E6 specific PCR-products (~700 bp) and expected size of mutation specific cleavage products (~350 bp for HPV18E6 and 200 bp and 500 bp for HPV16E6). Mutation rates calculated in percentages from the differences in band strength of the original PCR product and cleavage products are depicted below.

To prove CRISPR-mediated mutation induction at the target sites of the respective gRNAs, HeLa and SiHa cells were transduced with HPV18-E6 specific CRISPR-HCAdV or HPV16-E6 specific CRISPR-HCAdV, respectively. Then, 48 h post-transduction, genomic DNA of HPV18-E6-specific CRISPR-HCAdV-treated HeLa cells and HPV16-E6-specific CRISPR-HCAdV-treated SiHa cells was isolated for mutation detection. HPV-E6 loci surrounding the respective gRNA target sites were amplified by PCR. Resulting PCR amplicons were then subjected to hetero-duplex formation and digested with T7 endonuclease 1. Specific cleavage products of the expected size were detected for HPV18-E6 and HPV16-E6 respectively indicating successful mutation induction at the predicted target sites (Figure 2).

### 2.2. Decrease of HPV Tumor Cell Survival

**Figure 3:**
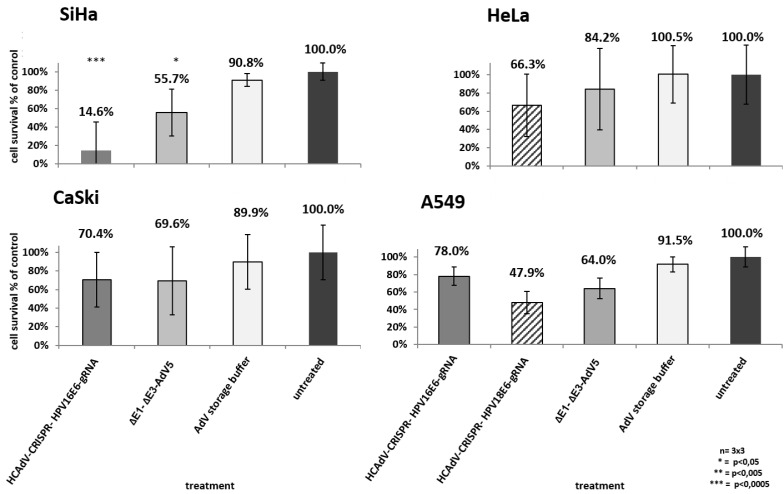
Monitoring of cell survival in SiHa, HeLa, CaSki, and A549 cells. Cells were transduced at confluency with HPV-E6-specific CRISPR/Cas9-HCAdV or ΔE1-ΔE3-AdV5 with 1000 infectious vector particles per cell. Cell viability was measured using a CCK-8 assay five days post-transduction. HPV-positive tumor cells showed reduced viability compared to untreated controls. Standard deviations of mean values are shown as error bars. Statistically significant differences compared to untreated controls are shown as one, two, or three stars indicating *p* values < 0.05, <0.005, and <0.0005 respectively.

To examine the potential of HCAdV armed with HPV-E6 specific CRISPR/Cas9 to reduce HPV tumor specific cell survival, we transduced HeLa, SiHa and CaSki cervical cancer cells as well as HPV negative A549 lung carcinoma cells with HPV18-E6 specific CRISPR-HCAdV, HPV16-E6 specific CRISPR-HCAdV, ΔE1-ΔE3-AdV5, or AdV storage buffer. Compared to untreated controls or AdV storage buffer treated controls, cervical carcinoma cells treated with HPV-E6-specific CRISPR-HCAdV showed a reduced number of surviving cells (Figure 3). SiHa cells transduced with HPV16-E6-specific CRISPR-HCAdV showed a significant reduction of 85.4% of surviving cells, whereas transduction with ΔE1-ΔE3-AdV5 led to a reduction of 44.3% of viable cells. CaSki cells transduced with HPV16-E6-specific CRISPR-HCAdV showed a reduction of 29.6% of metabolizing cells (Figure 3). In contrast, transduction with ΔE1-ΔE3-AdV5 resulted in a reduction of 30.4% of viable cells. In HeLa cells transduced with HPV18-E6-specific CRISPR-HCAdV showed a viability reduction of 33.7%, whereas transduction with ΔE1-ΔE3-AdV5 decrease viable cells of 15.8% (Figure 3). Even though a trend for reduction of cell viability can be observed CaSki and HeLa cells, values obtained for these cells were not statistically significant. For HPV-negative A549 cells, transduced with CRISPR expressing HCAdV, cell viability was also reduced. Transduction with HPV16-E6 specific CRISPR-HCAdV led to a reduction of 22% of viable cells and for HPV18-E6 specific CRISPR-HCAdV to a reduction of 52.1% of viable cells. ΔE1-ΔE3-AdV5 resulted in a reduction of 36% when measuring viable cells in A549 cells. Compared with untreated or buffer treated controls, values were not statistically significant (Figure 3).

Following the CCK-8 cell viability screening, the medium was removed, and cells were subjected to methylene blue staining to confirm the previous results using a different methodology that visualizes the healthy attached cells. The results of the methylene blue staining support the results obtained for the CCK-8-based viability assay and showed even stronger effects on the attachment of cells as quantified by the CCK-8 assay. In HeLa, SiHa, and CaSki, a clear decrease of attached cells could be seen after transduction with the respective vector at MOI 1000, whereas untreated controls (MOI 0) or AdV storage-buffer-treated controls were well attached (Supplementary Materials Figure S1). A549 cells showed reduction in cell attachment when treated with HPV18-E6 or HPV16-E6-specific CRISPR-HCAdV or ΔE1-ΔE3-AdV5 (Figure S1).

### 2.3. Cervical Cancer Cell Lines Show Different Susceptibility to AdV5

To find out whether the differences in the effect of the HPVE6 specific CRISPR/Cas9 expressing HCAdV on different cervical cancer cell lines is caused by different transduction efficiencies of the vector, we determined the susceptibility of SiHa, HeLa, and CaSki cells to AdV5. We infected each respective cell line with defined numbers of viral particles of a GFP-luciferase expressing E3 deleted AdV5.

**Figure 4:**
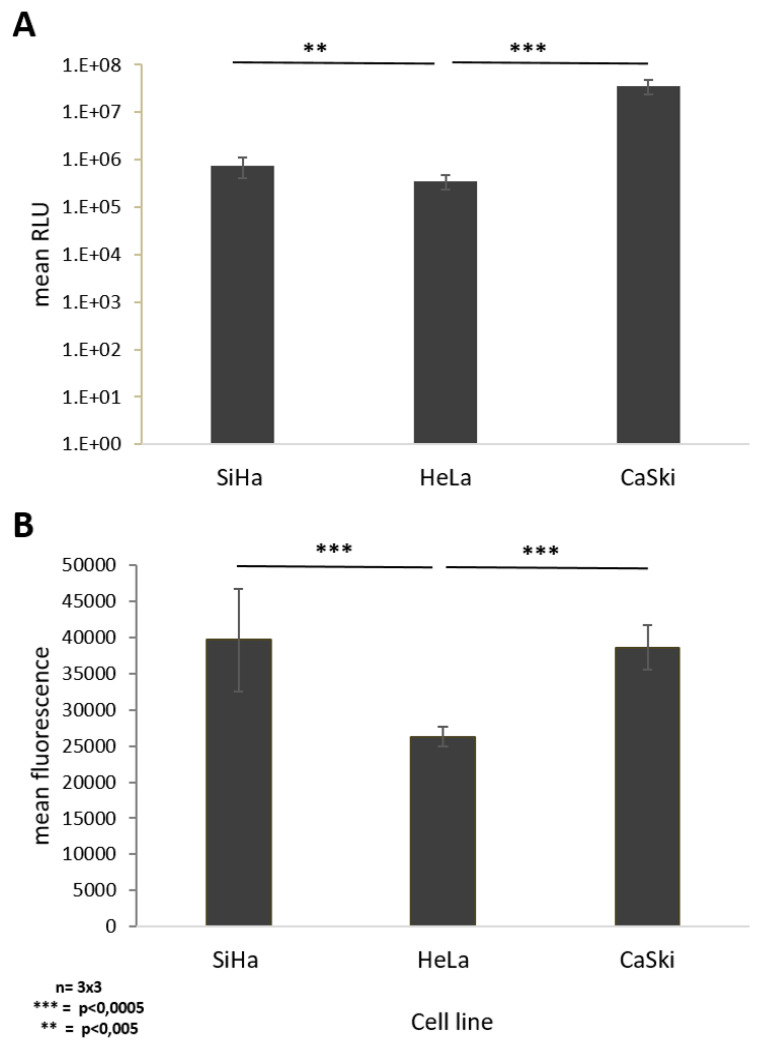
Monitoring cell susceptibility of SiHa, HeLa, and CaSki cells to AdV5. Siha, HeLa, and Caski cells were infected with E3-deleted AdV5-expressing GFP and luciferase at different doses. (**A**) AdV5 mediated luminescence 24 h post transduction with 20 viral particles per cell (vpc). (**B**) AdV 5 mediated fluorescence 48 h post transduction with 1000 vpc. Standard deviations of mean values are shown as error bars. The line above the columns indicate which sampled were compared to each other Statistically significant differences of the cell lines compared to each other are shown as two or three stars, indicating *p* values < 0.005, and 0.0005 respectively.

24 h post transduction with 20 viral particles per cell, quantification of luciferase activity of transduced cells showed a significant 100.4-fold increase in luminescence in SiHa cells compared to CaSki cells, whereas HeLa cells revealed a 2.1-fold increase in luciferase expression levels compared to CaSki cells (Figure 4A). At low virus concentration, SiHa cells seem to be more susceptible to AdV5 infection than HeLa and CaSki cells (Figure 4A).

Due to saturation of the luminescence signal at higher viral particle numbers, we compared susceptibility of the different cell lines to AdV5 by quantifying the fluorescent signal from vector-derived GFP expression. Quantification of the mean fluorescence intensity 48 h post transduction of each respective cell line with 1000 viral particles per cell showed a significant 1.5-fold increased fluorescence signal in SiHa and HeLa cells if directly compared to CaSki cells, respectively. No difference was observed between SiHa and HeLa cells (Figure 4B).

### 2.4. Reduction of Proliferation of HPV Positive Cancer Cell Lines

**Figure 5:**
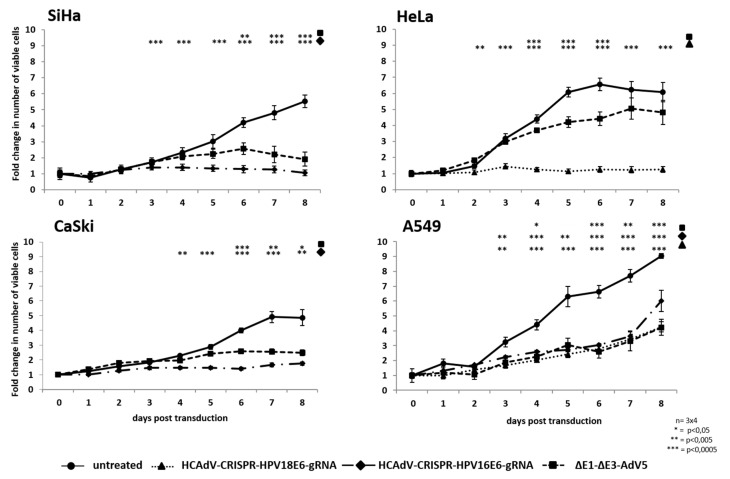
Monitoring of cell proliferation in SiHa, HeLa, CaSki, and A549 cells. Following transduction with the respective HPV-E6-specific CRISPR/Cas9-HCAdV or ΔE1-ΔE3-AdV5 with 1000 infectious vector particles per cell, cell proliferation was monitored for eight days. The relative number of viable cells at each time point post-transduction was measured by CCK-8 assay. Standard deviations of mean values are depicted as error bars. Statistically significant differences compared to untreated controls are shown as one, two or three stars, indicating *p* values < 0.05, <0.005, and <0.0005 respectively.

To investigate whether HPV-E6 specific CRISPR-HCAdV can reduce proliferation of HPV-induced cervical cancer cells, we transduced HPV18 containing HeLa cells, HPV16-positive SiHa and CaSki and SiHa cervical cancer cells as well as HPV-negative A459 lung carcinoma cells. We applied the vectors HPV18-E6 specific CRISPR-HCAdV, HPV16-E6 specific CRISPR-HCAdV or ΔE1-ΔE3-AdV5 at MOI 1000 and monitored the increase of viable cells for eight days. Transduction with HPV16-E6-specific CRISPR-HCAdV inhibited cell proliferation of SiHa cells and the number of viable cells significantly differed from untreated controls already three days post-transduction. In contrast, transduction with ΔE1-ΔE3-AdV5 only led to a significant reduction of cell proliferation that was significantly different from untreated controls after day 6 (Figure 5). Transduction with HPV16-E6 specific CRISPR-HCAdV inhibited cell proliferation of CaSki cells and the number of viable cells was significantly reduced compared to untreated controls already four days post-transduction. Transduction with ΔE1-ΔE3-AdV5 also resulted in a significant reduction of cell proliferation that was significantly different from untreated controls after day 6 (Figure 5). Transduction with HPV18-E6 specific CRISPR-HCAdV strongly inhibited cell proliferation of HeLa cells, which was in sharp contrast to untreated controls already three days post-transduction. Transduction with ΔE1-ΔE3-AdV5 resulted in a less pronounced reduction of cell proliferation that was still significantly different from untreated controls between days 4–6 (Figure 5).

Note that transduction with HPV16-E6-specific CRISPR-HCAdV, HPV18-E6-specific CRISPR-HCAdV, or ΔE1-ΔE3-AdV5 also reduced cell proliferation of A549 cells, which was different from untreated controls. Even though cell proliferation was reduced proliferation continued until the end of the experiment independent of the treatment (Figure 5). Compared to untreated control cells, treatment with HPV-E6 specific CRISPR-HCAdV or ΔE1-ΔE3-AdV5 led to a decreased cell proliferation in HPV-induced tumor cells. In HeLa cells, cell proliferation was strongly inhibited by the HPV18-E6-specific CRISPR-HCAdV and ΔE1-ΔE3-AdV5. In SiHa and CaSki cells, HPV16-E6-specific CRISPR-HCAdV and HPV18-E6-specific CRISPR-HCAdV and ΔE1-ΔE3-AdV5 reduced cell proliferation when compared to untreated cells. In contrast, HPV negative A549 cells were almost unaffected by HPV-E6-specific CRISPR-HCAdV when compared to untreated controls. As seen in HPV-positive cells ΔE1-ΔE3-AdV5 was also able to inhibit cell proliferation in HPV-negative A549 cells. Taken together, these results suggested that tumor cell proliferation inhibition of specific HPV-E6-specific CRISPR-HCAdV is specific for HPV-positive cervical cancer cells and that ΔE1-ΔE3-AdV5 acts through a different mechanism possibly conditional replication in tumor cells.

### 2.5. Vector Treatment Lead to Increase in p53 Protein Levels

**Figure 6:**
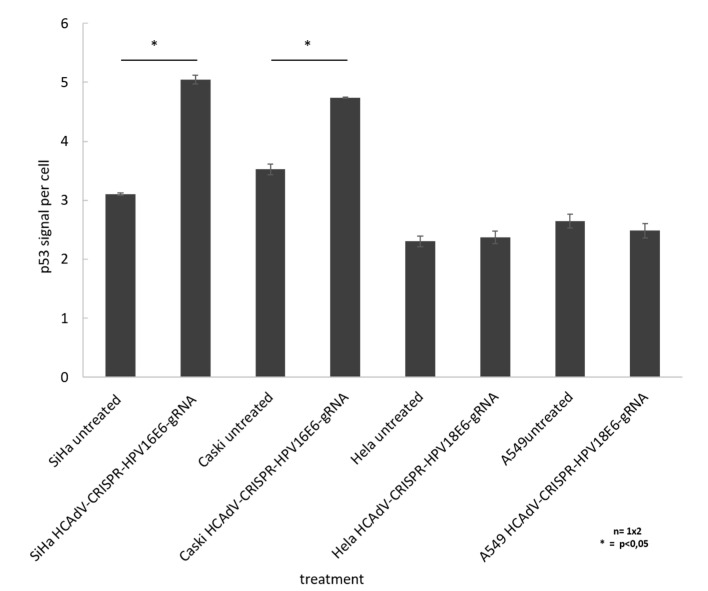
Monitoring change in p53 levels in treated SiHa, HeLa, and CaSki cells. In cell western analyses based on p53 quantification of cells treated with MOI 1000 of respective vectors compared to untreated controls 96 h post transduction. For each well, the fluorescent intensity values of the p53 antibody measurement at 800 nm was divided by the mean fluorescent intensity of cell tag700 at 700 nm to normalize for the cell number (p53 signal per cell). Standard deviations of mean values are shown as error bars. Statistically significant differences compared to untreated controls are shown as one star, indicating *p* values < 0.05.

To prove that HCAdV delivery of HPV16E6 or HPV18-specific CRISPR/Cas9 and subsequent E6 mutagenesis increased apoptosis in a p53 dependent manner, we performed cell western analysis to quantify the change in cellular p53 level upon vector transduction. The results show a significant of 1.6-fold and 1.3-fold increase of p53 for SiHa and CaSki cells, respectively, when treated with HCAdV-CRISPR-HPV16E6gRNA. HeLa and A549 cell treated with HCAdV-CRISPR-HPV18E6gRNA showed no change in p53 levels (Figure 6).

### 2.6. Increase in Apoptosis Induction

**Figure 7:**
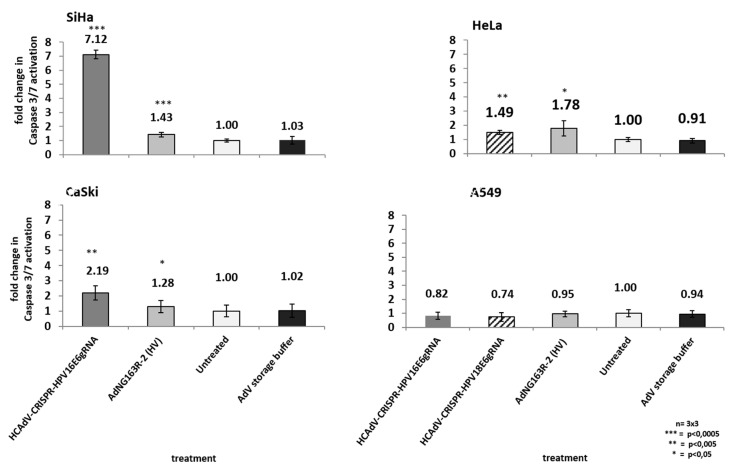
Monitoring apoptosis induction in SiHa, HeLa, CaSki, and A549 cells. Here, 48 h post-transduction with the respective HPV-E6-specific CRISPR/Cas9-HCAdV and ΔE1-ΔE3-AdV5 at 1000 infectious vector particles per cell, caspase 3/7 activation was measured. Standard deviations of mean values are shown as error bars. Statistically significant differences compared to untreated controls are shown as one, two, or three stars, indicating *p* values of <0.05, <0.005, and <0.0005 respectively.

As high-risk HPV-E6 proteins interact with the cellular p53 tumor suppressor and mediate its proteasomal degradation, HPV-induced cervical cancer cells can circumvent p53 mediated apoptosis induction. Inactivation of HPV-E6 should inhibit HPV-E6-mediated p53 degradation, leading to a re-accumulation p53 protein and a reactivation of p53 mediated apoptosis induction. To investigate whether HCAdV armed with HPV-E6-specific CRISPR/Cas9 specifically induce cell death in HPV tumor cells by mediating apoptosis induction, we transduced HPV18-containing HeLa cells, SiHa and CaSki cervical cancer cells, and A459 lung carcinoma cells. We applied the vectors HPV18-E6-specific CRISPR-HCAdV, HPV16-E6-specific CRISPR-HCAdV, and ΔE1-ΔE3-AdV5. Two days post-transduction, the increase of apoptosis induction was determined by measuring the activation of effector Caspases 3/7. Compared to untreated controls, transduction of SiHa cells with HPV16-E6 specific CRISPR-HCAdV significantly increased Caspase 3/7 induction (7.1-fold), whereas transduction with ΔE1-ΔE3-AdV5 only increased Caspase 3/7 induction 1.4-fold (Figure 7). Transduction of CaSki cells with HPV16-E6-specific CRISPR-HCAdV significantly increased Caspase 3/7 induction 2.19-fold and transduction with ΔE1-ΔE3-AdV5 increased Caspase 3/7 induction 1.28-fold (Figure 7). Transduction of HeLa cells with HPV18-E6 specific CRISPR-HCAdV increased Caspase 3/7 induction 1.49-fold, whereas transduction with ΔE1-ΔE3-AdV5 significantly increased Caspase 3/7 induction 1.78-fold (Figure 7). Transduction of A549 cells with HPV16-E6-specific CRISPR-HCAdV, HPV18-E6-specific CRISPR-HCAdV, or ΔE1-ΔE3-AdV5 moderately reduced Caspase 3/7 induction but without any statistical significance.

## 3. Discussion

Previous studies demonstrated that designer-nuclease-mediated destruction of HPV oncogenes are efficient strategies to combat HPV-induced tumors by inducing apoptosis [26,27,28,29,30,41]. To exploit this strategy as a therapeutic intervention, we aimed at translating the findings based on CRISPR/Cas9-mediated HPV oncogene destruction toward a potential in vivo delivery strategy using an all-in-one CRISPR-HCAdVs containing a Cas9 expression cassette and a HPV E6 specific gRNA expression. We tested the effects of the HPVE6 specific CRISPR/Cas9 expressing HCAdV on viability, proliferation and apoptosis induction of cervical cancer cell lines compared to a ΔE1-ΔE3-AdV5-CRAdV that kills tumor cells but not non-transformed cells due to the cytopathic effect caused by AdV replication [42,43,44,45,46,47]. The results of this study prove that HCAdV can be used as delivery vehicles for the HPV-E6-specific CRISPR/Cas9 machinery. Transduction of three different HPV-induced cervical cancer cell lines HeLa, CaSki, and SiHa with HPV-oncogene-specific CRISPR-HCAdV inhibited tumor cell proliferation and increased cell death though induction of apoptosis.

Noteworthy, even though the same cell numbers were infected with the same MOI, the antitumor effects in the cell lines investigated in this study differ. In SiHa cells, the antitumor effects of our vector treatment were strong, whereas CaSki cells showed no or at least weak response to the treatment (Figure 3, Figure 5, Figure 6 and Figure 7). The response to vector treatment in part reflects the different susceptibility of the studied cell lines to the vector. When treated with low viral particle numbers, SiHa cells are 100-fold more susceptible to AdV5 than CaSki cells (Figure 4A). In contrast, at high viral particle numbers per cell, the difference in vector susceptibility is less pronounced. HeLa and SiHa cells are 1.5-fold more susceptible than CaSki cells with no differences between SiHa and CaSki cells (Figure 4B). Therefore, differences in vector susceptibility only partially explain the differences in response to vector treatment.

Another difference between the cervical cancer cells studied here is the genome copy number of HPV genomes integrated into the chromosomes. Those differences might also contribute to the differences in response to vector treatment. SiHa cells contain 1–2 HPV16 genome copies integrated into their chromosomes, whereas CaSki cells contain more than 600 copies of HPV16 in 11 chromosomal sites, as well as low copy numbers of partial HPV18 genomes [48,49,50,51]. The intermediate response in HeLa cells to the vector treatment might also be explained by the intermediate number of HPV18 genomes [52] in HeLa cells. HeLa cells contain at least five copies of the HPV18 genome [53,54]. Therefore, the difference in response to the vector treatment could be explained by a large number of uncut HPV copies remaining within HeLa and even more in CaSki cells leading to ongoing anti-apoptotic E6 effects within these cells. In SiHa cells, CRISPR/Cas9 seemed to have mutated enough E6 sequences on genome level to inhibit E6 effects. HPV copy numbers in cervical tumors depends on the integration frequencies and chromosome duplication events and can vary between different lesions. As HPV integration status and copy number seem to influence the effect of an anti-HPV-CRISPR approach, molecular diagnostics determining the HPV type, integration status, and viral load, can be predictive for the efficiency of such an anti-HPV-CRISPR HCAdV treatment. As we observed differences in the susceptibility of the different cells to the vector and differences in response to the treatment, pretreatment diagnostics could also help predict the optimal vector dose. This strategy enables to identify the optimal dose to achieve the desired response in a cell line-dependent manner and in the future to develop a personalized therapy approach for patients.

It remains unclear why the cell survival of HPV negative A549 cells was affected after transduction with HPV oncogene-specific CRISPR-HCAdVs. All used vectors including the ΔE1-ΔE3-AdV5 non-CRISPR control influenced cell viability in A549 cells. This is probably due to the high transduction efficiencies of adenoviral vectors in A549 cells. It is well established that A549 cells are highly susceptible to adenovirus infection. As all vectors negatively influenced A549 cell survival and proliferation this is probably not due to the genetic cargo, but rather a general reaction to the viral transduction. Zhen et al. [26,29], as well as Kennedy et al. [28], who published the gRNAs used in this study, did not report any off-target effects after plasmid transfection. As cellular toxicity due to Cas9 overexpression has been reported in hematopoietic stem cells [55], the presence of Cas9 protein could have led to decreased cell viability of A549 cells. Before further developing anti-HPV-CRISPR HCAdV treatment, further studies including other HPV-negative control cell lines and especially control vectors containing a non-specific gRNA or vectors containing no gRNA are needed to elucidate whether decreased viability in HPV-negative cells is due to the vector treatment, the presence of Cas9 and potential unspecific action, or an artifact resulting from the assay used to quantify viable cells.

Compared to the CRISPR-expressing HCAdVs, ΔE1-ΔE3-AdV5 showed slightly stronger inhibitory effects on cell viability. However, HPV-specific CRISPR/Cas9-expressing HCAdV led to much stronger Caspase 3/7 induction in HPV-positive cells. However, in A549, no significant induction of apoptosis was observed, indicating that the inhibitory effects on viability and proliferation of HPV positive cells were specific to the CRISPR/Cas9-mediated E6 disruption and induction of apoptosis. Previous studies have shown that transfection of HPV-positive cervical cancer cells with HPV-specific CRISPR/Cas9 expression plasmids lead to increased p53 levels [26,30]. Following adenoviral delivery of HPV specific CRISPR/Cas9 we also observed an increase in p53 levels (Figure 6), indicating that the elimination of HPV oncogenes leads to a re-accumulation or less p53 degradation, which explains the higher number of cells that showed increased Caspase 3/7 activity upon vector treatment (Figure 7).

HCAdV armed with HPV-E6-specific CRISPR/Cas9 drive cell death and inhibition of cell proliferation of HPV-positive cervical cancer cells through apoptosis induction, whereas ΔE1-ΔE3-AdV5 did not significantly increase apoptosis induction in HPV-positive or HPV-negative cell lines. The observed ΔE1-ΔE3-AdV5-mediated tumor cell killing is apoptosis independent and could rather be explained as a result of viral replication. This shows that the effects seen after transduction with HPV-specific CRISPR/Cas9-expressing HCAdV are related to the specific effects of it CRISPR/Cas9 cargo rather than the transduction process or potential replication of remaining helper virus particles within the HCAdV preparations. The versatility of the CRISPR/Cas9 system in combination with HCAdV delivery which can be constructed in a facilitated way through our CRISPR-HCAdV production pipeline [38] offers the possibility to develop personalized treatment depending on the causative HPV type present in a respective patient tumor. Here, precise diagnostics of the HPV type and viral load within the tumor will help to choose the optimal treatment regimen.

Translation of the present results towards preclinical in vivo models is challenging. Xenograft models in immune-deficient mice or transgenic mice expressing HPV oncogenes are a considerable model system that can be exploited. Here, the route of administration will be of particular interest. Systemic injection of AdV vectors based on AdV serotype 5 will efficiently transduce the liver [56], limiting the effectiveness for treating epithelial tumors. Regarding the necessity to treat epithelia with precursor lesions, invasive tumors and infected areas without transformation in close proximity [5], alternative strategies should be considered. As AdVs are able to penetrate the upper layers of epithelium of the mouse skin without inducing cytotoxic effects [57], vector delivery through microporation [58,59] could be a relevant option.

Besides the route of administration modification of the vectors used for this approach can contribute to optimize delivery to relevant cells. Chemical modification of vector capsids [60] or using Darpins as adaptors between target cells and vector [61] could direct the vectors to the desired cell type to increase specificity and efficiency of the approach. Recently, an alternative AdV serotype became available [62,63,64]. Vectorization of such serotypes could contribute to an improved efficiency of the approach.

However, the setup of transgenes used in the HCAdV-expressing HPV-specific CRISPR/Cas9 used in this study does not exploit the full antitumor potential of what HCAdV could achieve. Additional gRNAs targeting could be included to enhance the anti-HPV effects. Addition of RNA interference directed against HPV oncogene mRNA [14,18] could be added to reach synergistic effects. A combined expression of immune modulators [65] or anti-neoangiogenic factors [66] could also be considered. Arming CRAdV, with HPV oncogene specific CRISPR/Cas9 could enhance efficacy through synergistic effects. Therefore, combining the effects of HPV-oncogene-specific designer nucleases with oncolytic virotherapy is also a reasonable strategy that deserves further commitment.

## 4. Materials and Methods

### 4.1. Viral Vectors

An E1and E3-deleted, first-generation Adenovirus AdNG163R-2 (ΔE1-ΔE3-AdV5) was produced as previously described [37]. HPV16 and HPV18-specific CRISPR/Cas9-expressing HCAdV based on human AdV serotype 5 (AdV5) were produced as previously described [38]. In brief, HCAdV-CRISPR-HPV16E6gRNA and HCAdV-CRISPR-HPV18E6gRNA contained a staphylococcus pyogenes Cas9 gene driven by a CbH promoter and a gRNA expression unit controlled by human U6-RNA promoter. HCAdV-CRISPR-HPV18E6gRNA contained an additional GFP expression cassette driven by CMV promoter (Figure 2). The gRNA sequences for HPV18-E6 (GCGCTTTGAGGATCCAACA) and HPV16-E6 (CAACAGTTACTGCGACGTG) were previously published [26,29].

Quantification of infectious virus particles within the vector preparations was carried out as previously described [67,68]. Briefly, HEK293 cells were transduced with defined volumes of the purified vectors. Three hours post transduction cells were washed with 1x phospahate-buffered saline (PBS) prior to harvesting and harvested cells were centrifuged and resuspended in 1x PBS to remove non-infective particles and centrifuged again. Following isolation of genomic DNA from transduced cells, HCAdV vector genome copy numbers were determined by HCAdV-specific q-PCR.

### 4.2. Cell Lines and Cell Culture

The cervical cancer cell lines SiHa and CaSki containing HPV16 and HeLa-containing HPV18 genomes integrated into their chromosomal DNA as well as HPV-negative A549 lung carcinoma cells were cultured in DMEM medium supplemented with 10% fetal bovine serum (FBS, PAN Biotec, Aidenbach, Germany), 100 U/mL Penicillin and 100 µg/mL Streptomycin (PAN Biotec) unless stated otherwise. Cells were grown at 37 °C and 5% CO_2_.

### 4.3. Mutation Induction and Detection

Determination of genome targeting efficiency using T7 Endonuclease I was performed using a protocol adapted from previously published protocols [69]. Briefly, 2 × 10^6^ cells were seeded into 24-well plates and subsequently infected with the respective HPV type-specific CRISPR-HCAdVs. Then, 48 h post-infection, genomic DNA was extracted from the cells for subsequent PCR using a Nucleo Spin Tissue kit (Macherey-Nagel, Düren, Deutschland). PCR amplicons covering the respective HPV-E6 region surrounding the respective gRNA binding sites was amplified using Phusion high fidelity DNA polymerase kit (New England Biolabs, Frankfurt, Germany) according to manufacturer’s instructions. The HVP18-E6 amplicon was generated using the primer-set HPV18T7E1fwd (5′ CTTGCATAACTATATCCACTCCC 3′) and HPV18E6rev (5′ ATTCAACGGTTTCTGGCAC 3′) yielding a PCR product of 656 bp and HPV16E6 region was amplified using the primer-set HPV16E6fwd (5′ TGAACCGAAACCGGTTAGTA 3′) and HPV16E6rev (5′ TGAACCGAAACCGGTTAGTA 3′) (30) yielding a PCR product of 660 bp. PCR products were purified by ethanol precipitation by filling the PCR volume up to 100 µL with H_2_O, adding of 10 µL NaAc (3M, pH 8.0) and 250 µL ice cold EtOH (100%) followed by centrifugation for 10 min, high speed. The DNA-pellet was washed with 500 µL EtOH (70%) and centrifuged for 5 min at high speed. After centrifugation, the supernatant was discarded, and the pellet was air dried and resuspended in 17.5 µL H_2_O. Purified amplicons were supplemented with 2µL of Buffer NEB2 (New England Biolabs, Ipswich, MA, USA) and subjected to heteroduplex formation by heating them to 95 °C and ramping down to room temperature using a cooling rate of 0.1 °C/s. After heteroduplex formation, 0.5 µL of T7E1 enzyme (New England Biolabs) was added and the mixture was incubated for 15 min at 37 °C. The reaction was stopped by adding purple loading dye (New England Biolabs, containing 10 mM EDTA, 0.08% SDS) and separated on a 2% agarose gel and at ~90 V for >45 min. Mutation rate was calculated according to the formula: % gene modification = 100 × (1 – (1 − fraction cleaved)1/2) [69].

### 4.4. Cell Viability Assay and Crystal Violet Staining

We seeded 1.5 × 10^6^ HeLa, CaSki, SiHa, and A549 cells into 24-well plates. Five hours later, cells were transduced with 0, 0.1, 1.0, 10, 100, or 1000 infectious particles of the respective HPV16 or HPV18-specific CRISPR/Cas9-expressing HCAdV or AdNG163R-2. To rule out potential cytotoxic effects of the solvent used to dilute the vectors, negative controls cells were treated with serial dilution of AdV storage buffer (50 mM Hepes pH 7.4, 100 mM NaCl, 10% Glycerol), equaling the highest volume of buffer that was applied for MOI 1000 of the HPV16-specific CRISPR/Cas9-expressing HCAdV. Five days post-transduction, cell-viability was determined using the Cell Counting Kit-8 (CCK-8; Sigma, St. Louis, MS, USA) following manufacturer’s instructions. Briefly, 10µL of CCK8 substrate were applied to each well containing 100 µL of cell culture medium and incubated for 1 h to allow viable cells to convert the CCK-8 substrate into a colored dye. Adsorption at 450 nm was measured using a GENios multi-plate reader (TECAN, Crailsheim, Germany). After CCK-8 readout, the medium was removed by three consecutive washing steps with 1 × PBS and fixed in 10% formaldehyde in PBS for 10 min at room temperature, followed by three consecutive washing steps with 1 × PBS. Adherent cells were stained with crystal violet staining solution for 10 min. Excess staining solution was removed by gently washing the plates in tap water. Subsequently plates were air dried and kept for photo documentation. All experiments were performed three times with triplicates for each sample.

### 4.5. Determination of Cellular Susceptibility to Human AdV Serotype 5 (AdV5)

To determine the susceptibility of the cell lines used in this study by means of GFP reporter gene expression, 40,000 cells of HeLa, SiHa, or CaSki cells were seeded in a 96-well plate. Then, 5 h post-seeding, cells were infected with 1000 viral particles of an E3 deleted AdV5-expressing GFP and luciferase that was produced as previously described [62]. Forty-eight hours post-transduction, virus-mediated GFP fluorescence within transduced cells was quantified using an Infinity 200 pro multi-plate reader (TECAN). To determine the susceptibility of the cell lines used in this study by means of luciferase reporter gene expression, 40,000 cells of HeLa, SiHa, or CaSki cells were seeded per well in a 96-well plate. Five hours post-seeding, cells were infected with 20 viral particles of an E3 deleted AdV5 expressing GFP and luciferase. Then, 24 h post-transduction, quantification of virus-mediated luciferase activity of transduced cells was carried out using the dual-luciferase reporter assay (Promega, Walldorf, Germany) following the manufacturer’s instructions. Luminescence measurement was performed using an Infinity 200 pro multi-plate reader (TECAN). Experiments were repeated three times in triplicates.

### 4.6. Proliferation Assay

We seeded 1 × 10^3^ HeLa, CaSki, SiHa or A549 cells per well into 96-well plates, respectively, and transduced with 1000 infectious vector particles per cell of the HPV16 or HPV18 specific CRISPR/Cas9 expressing HCAdV or AdNG163R-2, respectively. The number of viable cells for each treatment was determined using the Cell Counting Kit-8 (CCK-8; Sigma, St. Louis, MS, USA) in 24 h intervals in quadruplicates for seven days. All experiments were performed three times with quadruplicates for each sample.

### 4.7. In Cell Western Analysis of Cellular p53

To analyze changes in cellular p53 protein levels in response to HPV-specific CRISPR/Cas9-expressing HCAdV treatment, we performed in cell western analysis. Here, 25,000 cells of the respective cell line were seeded to each well of a 96 well plate. SiHa and CaSki cells were treated with HAdV-CRISPR-HPV16E6gRNA and Hela, and A549 cells were treated with HCAdV-CRISPR-HPV18E6gRNA at MOI of 1000, respectively. Controls were left untreated. Then, 96 h post-transduction, the medium was removed, and cells were fixed using 1× PBS, 4% paraformaldehyde for 20 min at room temperature. Fixed cells were washed three times using 1× PBS, 0.1% triton X100 for five minutes to permeabilize the cells. Following cell permeabilization, wells were incubated with blocking solution (LI-COR, Lincoln, NE, USA) for 90 min at room temperature. After blocking, wells were incubated with AlexaFluor790 coupled anti-p53 antibody (p53 (Do-1): sc-126; Santa Cruz, Dallas, Germany) and CellTag 700 Stain (LI-COR, Lincoln, NE, USA) diluted 1:50 and 1:500, respectively, in blocking solution for 150 min at room temperature. Background controls only received anti p53 antibody or cell tag 700, respectively. Finally, plates were washed with 1x PBS, 0,1% tween 20 for five times at room temperature before plates were air dryed and the 700 and 800 nm fluorescent signals of all wells were quantified using the Odyssey CLx scanner (LI-COR) and image studio 5.2 software (LI-COR). Measurements were carried out at a resolution of 169 µm using a focus depth of 3 mm. For each well, the fluorescent intensity values of the p53 antibody (800 nm) was divided by the mean fluorescent intensity of cell tag700 (700 nm) to normalize for the cell number. Data were analyzed in duplicates.

### 4.8. Apoptosis Assay

We seeded 2.5 × 10^4^ HeLa, CaSki, SiHa, or A549 cells into black-walled 96-well plates with a transparent bottom. Between experimental groups, one row was left empty to prevent the luminescent signal to influence neighboring wells. Five hours post-seeding, cells were transduced with HPV16 or HPV18-specific CRISPR/Cas9-expressing HCAdV or the E1-deleted, first generation Adenovirus (AdNG163R-2), respectively. To rule out potential apoptotic effects of the solvent used to dilute the vectors, untransfected cells were treated with AdV storage buffer (50 mM HEPES, pH 7.4, 100 mM NaCl, 10% Glycerol), equaling the highest volume of virus that was applied for MOI 1000 of the HPV16-specific CRISPR/Cas9-expressing HCAdV. Untreated cells were incubated with cell culture medium alone. Then, 48 h post-transduction, apoptosis induction was monitored by measuring caspase 3/7 activation using luminescent caspase 3/7 glow assay (Promega) following the manufacturer’s instructions. Luciferase signal indicating Caspase 3/7–mediated substrate cleavage was detected using a GENios multi-plate reader (TECAN). Cell culture medium alone or medium supplemented with the AdV storage buffer was measured on the same 96-well plate to determine background signal. These background values were subtracted from the sample values. All experiments were performed three times with quadruplicates for each sample.

### 4.9. Statistical Analysis

Statistical significance of differences between experimental groups and untreated controls were analyzed using Students t-test using the software GraphPad Prism 8 (San Diego, CA, USA). Significant differences with *p* values of *p* < 0.0005, *p* < 0.005, or *p* < 0.05 are depicted as ***, ** or * respectively.

## 5. Conclusions

In summary, this study shows a proof-of-concept for using the CRISPR/Cas9 technology delivered by the most advanced adenoviral vector (HCAdV) to treat HPV-derived tumors. We conclude that HCAdV can serve as HPV-specific cancer gene therapeutic agents when armed with HPV-type-specific CRISPR/Cas9. We believe that our approach can contribute to treatment options specific for the respective HPV type present in each individual tumor. The next step is the translation of this approach into relevant in vivo animal models and, in the long-term run, this approach may lead to a novel concept in personalized tumor therapy.

## Supplementary Materials

The following are available online at https://www.mdpi.com/2072-6694/12/7/1934/s1, Figure S1. Oncolysis assay to monitor cell viability.
