# Supplementary material for: Adenoviral Vectors Armed with PAPILLOMAVIRUs Oncogene Specific CRISPR/Cas9 Kill Human-Papillomavirus-Induced Cervical Cancer Cells
Source: Cancers (Basel). 2020 Jul 17;12(7):1934. doi: 10.3390/cancers12071934 (PMC7409089; doi:10.3390/cancers12071934)
Supplement: Supplementary file 1 [file cancers-12-01934-s001.pdf]

cancers

MDPI

*Supplementary Materials*

# Adenoviral Vectors Armed with PAPILLOMAVIRUs Oncogene Specific CRISPR/Cas9 Kill Human-Papillomavirus-Induced Cervical Cancer Cells

**Eric Ehrke-Schulz, Sonja Heinemann, Lukas Schulte, Maren Schiwon, and Anja Ehrhardt**

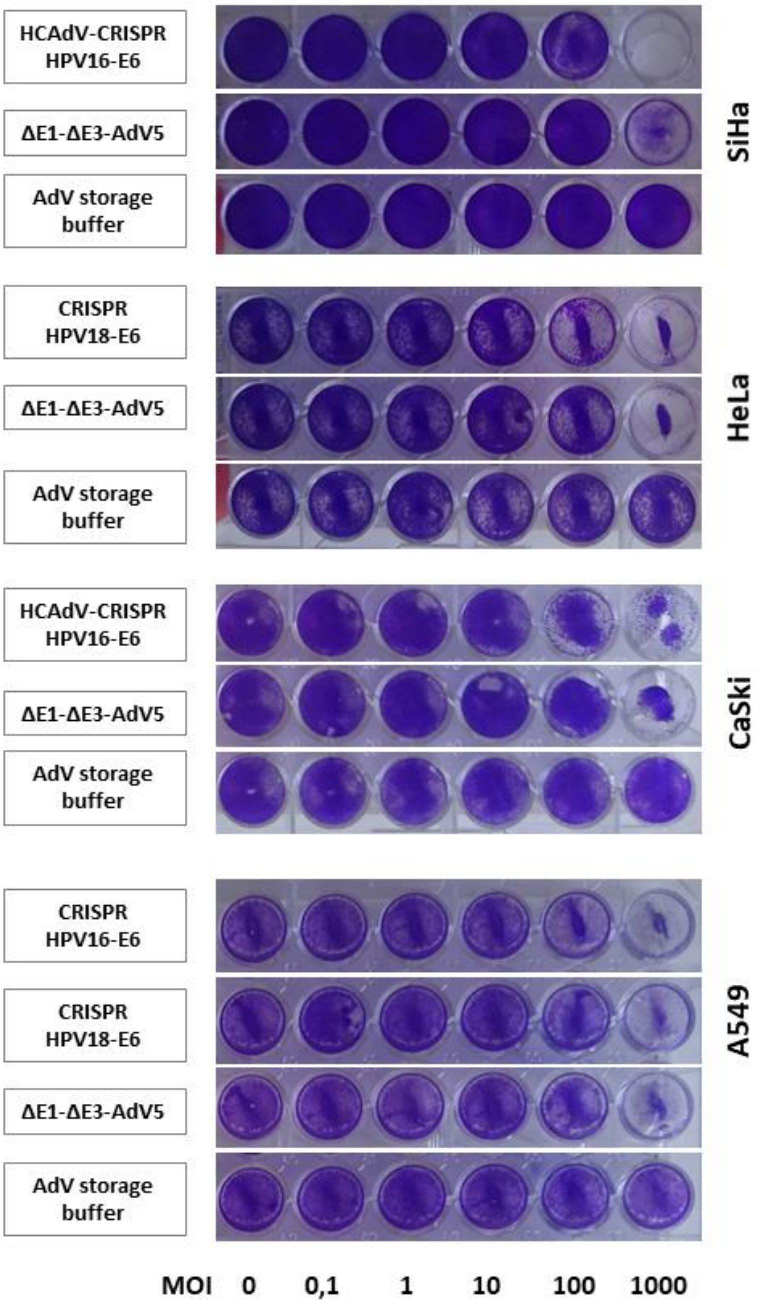


**Figure S1.** Oncolysis assay to monitor cell viability. Following transduction with HPV-E6 specific CRISPR/Cas9-HCAdV or AdNG163R-2 with increasing number of infectious vector particles per cell, HPV positive tumor-cells showed reduced viability compared to untreated controls at multiplicity (MOI) of 1000. AdV storage buffer treated cells were not affected.

© 2020 by the authors. Licensee MDPI, Basel, Switzerland. This article is an open access article distributed under the terms and conditions of the Creative Commons Attribution (CC BY) license (http://creativecommons.org/licenses/by/4.0/).
